# USE OF AND EXPERIENCES WITH ALTERNATIVE SITES FOR HEALTH CARE BY US ADULTS AGES 50–80

**DOI:** 10.1093/geroni/igae098.0085

**Published:** 2024-12-31

**Authors:** Teresa Keenan, Harriet Komisar, Olivia Dean, Erica Solway, Cheryl Lampkin

**Affiliations:** AARP, Washington, District of Columbia, United States; AARP, Washington, District of Columbia, United States; AARP, Oakland, California, United States; University of Michigan, Ann Arbor, Michigan, United States; AARP, Washington, District of Columbia, United States

## Abstract

In recent years, “alternative” sites for health care – such as urgent care and retail health clinics – have expanded to meet the growing demand for more convenient and accessible care. In August 2023, the University of Michigan National Poll on Healthy Aging asked a national sample of 2,687 adults ages 50-80 (completion rate: 50%) about their use of and opinions about alternative sites of care. In all, 60% of adults ages 50-80 reported using an alternative site in the past two years: urgent care clinic (47%), retail clinic (28%), worksite clinic (9%), and mobile health clinic (5%). Use of these alternative sites was more common among those ages 50-64, women, and those with higher incomes. Among older adults who visited an alternative care site in the past two years, 91% reported having a primary care provider (PCP). Of those with a PCP, 71% contacted their PCP about their visit to an alternative site. The most common reasons reported for visiting an alternative site were to avoid the hospital emergency department (44%) or to get a vaccine, specific test or to get a health exam (35%). The majority (62%) said they were likely (11% very likely, 51% somewhat likely) to use an alternative site for health care in the future, including 43% who had not used in alternative site in the past two years. Poll findings suggest that these sites will continue to play a significant role in the delivery of health care in the future.

